# AI-enabled precision evaluation of adrenal masses: radiomics, deep learning, and explainable imaging biomarkers

**DOI:** 10.3389/fendo.2026.1900157

**Published:** 2026-07-30

**Authors:** Chaohui Liang, Hao Zhu

**Affiliations:** 1 Department of Radiology, Taizhou Maternal and Child Health Hospital, Taizhou, Zhejiang, China; 2 Department of Radiology, The Third Affiliated Hospital of Wenzhou Medical University, Wenzhou, Zhejiang, China

**Keywords:** adrenal mass, artificial intelligence, deep learning, differential diagnosis, pheochromocytoma, radiomics

## Abstract

Accurate evaluation of adrenal masses remains a significant challenge in endocrinology and radiology, as differential diagnosis involves a wide spectrum of benign and malignant lesions. Radiomics and deep learning (DL) have emerged as promising tools to enhance the precision of adrenal mass assessment by extracting high-dimensional imaging features and enabling automated, data-driven analysis. This review summarizes the latest advancements in the application of radiomics and DL techniques for adrenal mass evaluation. We systematically describe the workflow of radiomic feature extraction and model development, emphasizing their roles in differentiating key lesions such as pheochromocytomas/paragangliomas (PPGLs), adrenal cortical adenomas, and adrenal cortical carcinomas. Additionally, the utility of these approaches in genotype prediction and prognostic evaluation is highlighted. The review further explores the advantages and potential of DL, particularly convolutional neural networks (CNNs), in automated segmentation, feature learning, and end-to-end diagnostic frameworks. Finally, current challenges including technical limitations, clinical translation barriers, and future research directions are discussed, aiming to provide a theoretical foundation for constructing intelligent and precise adrenal mass evaluation systems.

## 1 Introduction

Adrenal masses, commonly encountered in clinical practice, represent a heterogeneous group of lesions ranging from benign, non-functioning adenomas (NFA) to hormonally active tumors such as pheochromocytomas/paragangliomas (PPGLs) and highly aggressive malignancies like adrenocortical carcinoma (ACC) (1). The increasing use of cross-sectional imaging modalities, particularly computed tomography (CT), has led to a surge in the incidental detection of adrenal lesions, termed adrenal incidentalomas (AIs), with a prevalence estimated between 4% and 10% in the general population (2). While the majority of these lesions are benign and non-functioning, a subset harbors malignant potential or endocrine activity that necessitates timely and accurate diagnosis to guide appropriate management. Traditional imaging evaluation relies heavily on morphological features such as size, shape, presence of necrosis, and CT attenuation values, with unenhanced CT attenuation thresholds (e.g., <10 Hounsfield units (HU)) serving as a key discriminator for benign adenomas (3). However, these conventional criteria often fall short in complex cases, particularly in differentiating lipid-poor adenomas from malignant tumors or in assessing the malignant potential of lesions with overlapping imaging characteristics. Furthermore, biochemical evaluation remains indispensable for detecting hormone excess, yet some tumors exhibit atypical or mixed secretory profiles that complicate clinical assessment (4).

In recent years, the advent of radiomics and deep learning (DL) has revolutionized the landscape of medical imaging by enabling the extraction and analysis of high-dimensional quantitative features from standard imaging data. Radiomics transforms medical images into mineable data by capturing subtle textural, shape, and intensity patterns imperceptible to the human eye, thereby providing a more comprehensive characterization of tumor heterogeneity and microenvironment (5). Studies have demonstrated that radiomic features derived from CT images can effectively discriminate between benign and malignant adrenal lesions, including the differentiation of adrenocortical adenomas from carcinomas with high diagnostic accuracy (6). Moreover, radiomics has shown promise in predicting tumor grade and mitotic activity in ACC, which are critical prognostic factors influencing therapeutic decisions (7). Beyond diagnosis, radiomic analysis has been explored as a predictive tool for treatment response, such as forecasting the efficacy of etoposide-doxorubicin-cisplatin (EDP) chemotherapy in advanced ACC, highlighting its potential role in personalized medicine (8).

Complementing radiomics, DL—particularly convolutional neural networks (CNNs)—has emerged as a powerful approach capable of automatically learning hierarchical imaging features directly from raw data without the need for manual feature engineering. DL models have been successfully applied to classify adrenal masses on CT images, achieving high sensitivity and specificity in distinguishing ACC from lipid-poor adenomas and pheochromocytomas, even in cases with indeterminate biochemical profiles. These models facilitate end-to-end workflows encompassing lesion segmentation, feature extraction, and classification, thereby enhancing diagnostic efficiency and reproducibility. Importantly, interpretable machine learning or radiomics models incorporating explainability techniques such as Shapley Additive Explanations (SHAP) have been developed to elucidate the decision-making process, fostering clinical trust and adoption (9).

Despite these advances, challenges remain in integrating radiomics and DL into routine clinical practice for adrenal mass evaluation. Variability in imaging protocols, small sample sizes, and heterogeneity in study designs contribute to inconsistent results and limit generalizability (6). Moreover, the complex biology of adrenal tumors, including diverse histological subtypes and variable hormone secretion patterns, necessitates multimodal approaches that combine imaging, biochemical, genetic, and clinical data for comprehensive assessment. The role of molecular imaging modalities such as positron emission tomography (PET) with various radiotracers further complements anatomical imaging by visualizing metabolic and receptor-based tumor characteristics, aiding in localization and functional characterization (10). Additionally, emerging insights into tumor biology, including mitochondrial dysfunction and autophagy modulation, underscore the need for integrative biomarkers that reflect underlying pathophysiology.

In this context, the convergence of radiomics and DL offers a promising avenue to overcome current diagnostic limitations by harnessing the wealth of imaging data and uncovering latent patterns associated with tumor behavior. This review aims to systematically synthesize the current state of knowledge regarding the application of radiomics and DL in adrenal mass evaluation, elucidate the technical principles underpinning these methodologies, and critically appraise their clinical utility and limitations. Furthermore, we will explore future directions, including the integration of multi-omics data, development of standardized imaging protocols, and prospective validation in multicenter cohorts, to pave the way for precision diagnostics and tailored management strategies in patients with adrenal tumors. Through this comprehensive overview, we aspire to provide clinicians and researchers with a robust framework to harness advanced imaging analytics for improved patient outcomes.

## 2 Traditional methods and challenges in adrenal mass evaluation

### 2.1 Conventional imaging evaluation criteria and their limitations

CT remains the cornerstone imaging modality for the evaluation of adrenal masses, primarily due to its widespread availability, rapid acquisition, and high spatial resolution. The conventional CT assessment of adrenal lesions relies heavily on morphological features such as size, shape, margins, presence of necrosis or calcification, and attenuation values measured in HU. A key diagnostic criterion is the unenhanced CT attenuation value: adrenal adenomas typically exhibit low attenuation (<10 HU) on non-contrast scans, reflecting their high intracellular lipid content, which is a hallmark of benignity. This threshold has been widely adopted to differentiate lipid-rich adenomas from non-adenomatous lesions, including metastases and ACC (11). However, approximately 30% of adrenal adenomas are lipid-poor, exhibiting attenuation values above 10 HU, which complicates their differentiation from malignant lesions. In such cases, contrast-enhanced CT with washout analysis is employed, where adenomas demonstrate rapid contrast washout (>60% absolute washout), whereas malignant lesions typically show slower washout kinetics. Despite this, overlap exists, particularly with atypical adenomas and lipid-poor adenomas, limiting specificity (12).

Magnetic resonance imaging (MRI), especially chemical shift imaging (CSI), serves as an important adjunct when CT findings are inconclusive. CSI exploits the differential resonance frequencies of fat and water protons to detect microscopic fat within adrenal lesions. Adenomas with intracellular lipid exhibit signal loss on out-of-phase images compared to in-phase images, providing a sensitive marker for benignity. This technique is particularly valuable for characterizing lipid-poor adenomas that are equivocal on CT (13). Additional MRI sequences, such as T2-weighted imaging and diffusion-weighted imaging, can provide further tissue characterization, although their diagnostic accuracy is variable.

**Figure 1:**
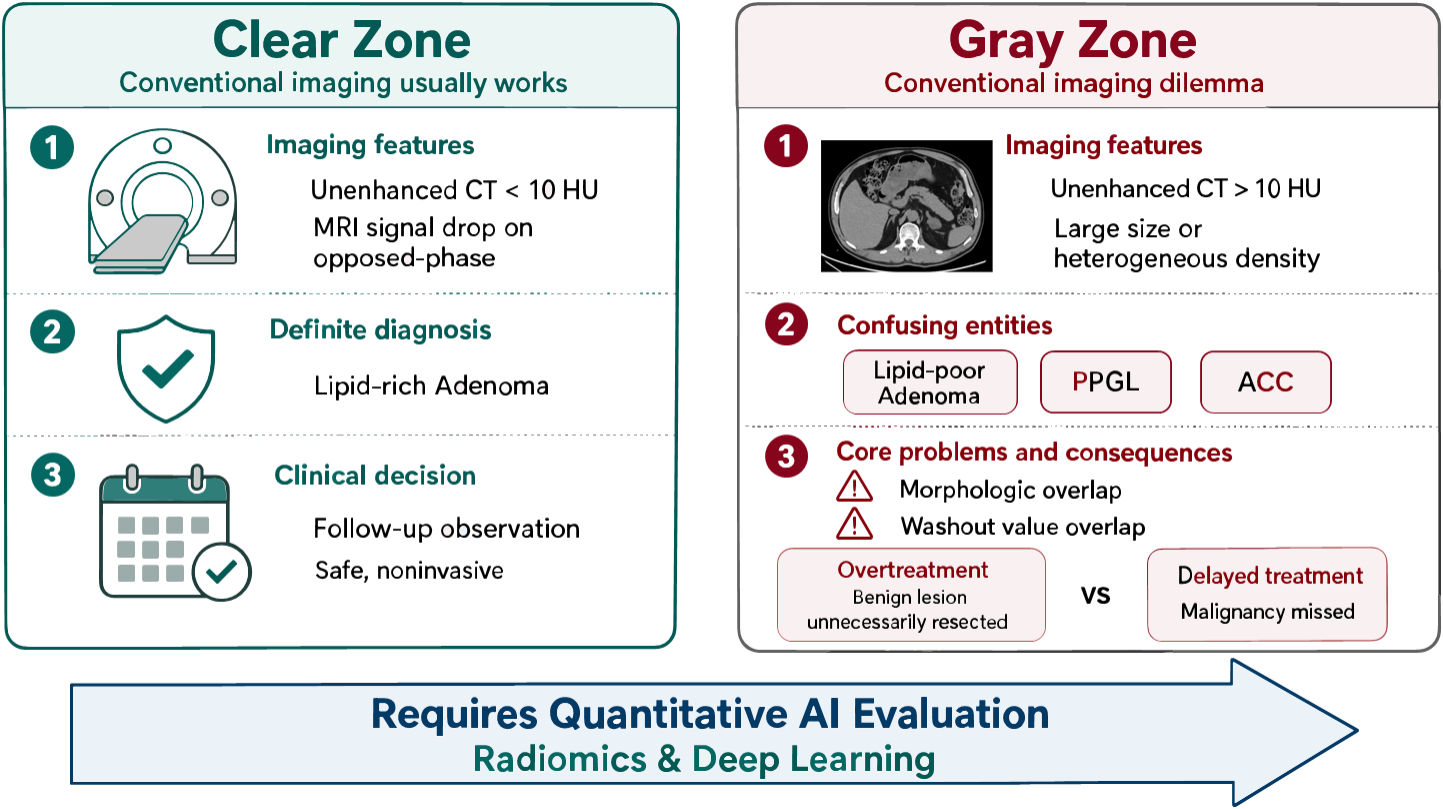
Diagnostic clear zone and gray zone in adrenal mass evaluation. Conventional imaging is effective for typical lipid-rich adenomas, but indeterminate adrenal lesions such as lipid-poor adenomas, pheochromocytomas/paragangliomas, and adrenocortical carcinoma may show overlapping imaging features. This diagnostic gray zone may lead to overtreatment or delayed treatment and supports the need for quantitative AI-assisted evaluation using radiomics and deep learning.

Nevertheless, these traditional imaging criteria face significant limitations. The sensitivity and specificity of CT and MRI in distinguishing atypical adenomas from malignant lesions such as metastases and ACC are suboptimal. For example, large adenomas (>4 cm), heterogeneous lesions, or those with necrosis and hemorrhage may mimic malignancy on imaging. Similarly, pheochromocytomas, which are neuroendocrine tumors of the adrenal medulla, can present with variable imaging features, sometimes overlapping with adenomas or carcinomas (11). Moreover, conventional imaging lacks the capability to predict genetic mutations or malignant potential, such as in pheochromocytomas with SDHx, VHL, or RET mutations, which influence tumor behavior and treatment strategies (14). This diagnostic “gray zone” often leads to uncertainty, resulting in unnecessary biopsies or surgeries, or conversely, delayed treatment of malignant lesions. The characterization of this clinical diagnostic dilemma, contrasting the straightforward “clear zone” of lipid-rich benign lesions with the overlapping “gray zone” of indeterminate masses and its associated clinical management consequences, is schematically illustrated in **Figure 1**. Thus, while CT and MRI remain foundational, their limitations underscore the need for advanced imaging biomarkers and integrative diagnostic approaches to improve accuracy in adrenal mass evaluation.

### 2.2 Clinical diagnostic challenges and unmet needs

The incidental discovery of adrenal masses, termed AIs, has become increasingly common with the widespread use of cross-sectional imaging. Clinicians face significant challenges in accurately distinguishing benign, NFA from lesions requiring intervention, such as subclinical pheochromocytomas, early-stage ACC, or metastatic tumors. This differentiation is critical because management strategies differ vastly, ranging from conservative monitoring to surgical resection. Traditional imaging and biochemical evaluations often fall short in this regard, leading to diagnostic dilemmas. For instance, lipid-poor adenomas and metastases may share overlapping imaging features, complicating non-invasive diagnosis and sometimes prompting unnecessary invasive procedures or delayed treatment (15, 16).

PPGLs represent a particularly heterogeneous group of adrenal tumors with diverse genetic backgrounds, including mutations in SDHx, VHL, and RET genes. These genotypes correlate with tumor location, metastatic potential, and therapeutic options. However, conventional imaging modalities lack the sensitivity to predict these genetic alterations or malignant potential preoperatively, limiting personalized treatment planning (14). Moreover, subclinical or biochemically silent pheochromocytomas may evade detection, posing perioperative risks due to catecholamine secretion if unrecognized.

Early diagnosis and prognostic stratification of ACC remain formidable challenges. ACC is a rare but aggressive malignancy with poor outcomes, often diagnosed at advanced stages due to nonspecific imaging features and lack of reliable biomarkers. Imaging criteria such as size and attenuation values provide some guidance, but overlap with benign lesions persists. Furthermore, conventional imaging cannot reliably predict tumor biology or response to therapy, underscoring the need for more precise, non-invasive biomarkers (17).

In addition to diagnostic challenges, there is an unmet clinical need for tools that can integrate multimodal data—imaging, clinical, biochemical, and genetic—to improve risk stratification and guide management. Current approaches often lead to either overtreatment, with unnecessary surgeries and biopsies, or undertreatment, with delayed intervention for malignant or hormonally active tumors. The heterogeneity of adrenal lesions, coupled with limitations of traditional imaging and biochemical tests, necessitates the development of advanced diagnostic methods, including radiomics and machine learning, to enhance accuracy, reduce invasive procedures, and enable personalized patient care (6, 18).

## 3 Technical foundations and applications of radiomics in the evaluation of adrenal masses

### 3.1 Radiomics analysis workflow and feature extraction

Radiomics analysis in adrenal mass evaluation follows a systematic workflow encompassing several critical steps: image acquisition and standardization, region of interest (ROI) segmentation, feature extraction and selection, and finally, model construction and validation. Initially, high-quality imaging data are acquired, typically via CT or MRI, with efforts made to standardize imaging protocols to reduce variability and enhance reproducibility across studies and centers (19, 20). Following acquisition, precise segmentation of the adrenal lesion is performed to delineate the ROI, which can be manual, semi-automatic, or fully automated using DL algorithms. Recent advances have demonstrated the feasibility and accuracy of automatic segmentation methods, such as two-stage cascade networks and 3D Res-Unet architectures, which achieve high Dice similarity coefficients and classification performance comparable to manual segmentation (16, 21). Accurate segmentation is crucial as it directly influences the quality of extracted features.

Once the ROI is defined, a comprehensive set of radiomic features is extracted. These features are categorized into first-order statistics, texture features, shape descriptors, and higher-order features derived from filtered images. First-order features quantify voxel intensity distributions within the ROI, including metrics such as mean, median, skewness, and entropy, reflecting the overall intensity characteristics of the lesion (22). Texture features capture spatial relationships and heterogeneity within the tumor, employing matrices such as the gray-level co-occurrence matrix (GLCM), gray-level size zone matrix (GLSZM), and gray-level run length matrix (GLRLM). These features provide insight into microstructural heterogeneity, necrosis, fibrosis, and vascular patterns that are not discernible by visual inspection alone (19, 23). Shape features describe the geometric properties of the lesion, including volume, surface area, sphericity, and irregularity, which can correlate with tumor invasiveness and growth patterns (22). Higher-order features are derived after applying filters such as wavelets or Laplacian of Gaussian, enhancing subtle image patterns and further characterizing tumor heterogeneity.

In adrenal masses, these radiomic features enable quantification of intratumoral heterogeneity, boundary irregularity, and microenvironmental complexity, which are linked to underlying pathological and molecular characteristics. For example, increased entropy and non-uniformity in texture features have been associated with malignant lesions and pheochromocytomas, reflecting their complex tissue architecture (22). The extracted features undergo rigorous selection processes, including statistical tests, least absolute shrinkage and selection operator (LASSO) regression, and correlation analyses, to identify the most informative and non-redundant features for model building (16, 24). Subsequently, machine learning or DL models are trained using these features, often combined with clinical parameters, to develop predictive tools for lesion characterization. Validation is performed internally and externally to assess model robustness, with performance metrics such as area under the curve (AUC), sensitivity, specificity, and F1 score reported (16, 19).

Overall, the radiomics workflow in adrenal lesion assessment integrates advanced image processing, quantitative feature extraction, and sophisticated modeling techniques to provide a non-invasive, reproducible, and objective approach to tumor characterization. This methodology surpasses traditional imaging evaluation by capturing subtle imaging biomarkers that reflect tumor biology, thereby enhancing diagnostic accuracy and aiding clinical decision-making. To visually clarify the implementation of these technical steps, **Table 1** comprehensively delineates the common software packages, mathematical feature categories, selection algorithms, and quality assessment methodologies utilized across current adrenal radiomics workflows.

**Table 1:** Common software packages, feature categories, and methodological standards in adrenal radiomics workflows.

Workflow step	Key common instruments & algorithms	Representative references	Clinical & technical significance in adrenal evaluation
ROI Segmentation	3D Slicer, LIFEx, nnU-Net, 3D Res-Unet	doi:10.1007/s00261-025-05345-5 doi:10.1080/07853890.2025.2540596 doi:10.3389/fendo.2023.1265790 doi:10.3389/fonc.2025.1619341 doi:10.1007/s10278-024-01158-y	Transitioning from labor-intensive manual contouring to reproducible, high-throughput automated 3D delineation to eliminate intra-observer bias.
Feature Extraction Software	PyRadiomics, TexRAD, LIFEx, SOPHIA, SIBEX	doi:10.3390/ijms23020637 doi:10.3389/fradi.2025.1635425 doi:10.1007/s40618-024-02476-2 doi:10.1186/s12880-023-01106-2 doi:10.3390/jimaging12040151 doi:10.3390/life15040560 doi:10.3389/fonc.2025.1619341	Extracting mineable data adhering to IBSI (Image Biomarker Standardization Initiative) standards to ensure feature cross-platform reproducibility.
Extracted Feature Categories	First-order statistics (Entropy, Skewness); Texture matrices (GLCM, GLSZM, GLRLM); Shape descriptors; Higher-order filters (Wavelet, LoG)	doi:10.3390/ijms23020637 doi:10.1093/bjsopen/zraa061 doi:10.3389/fradi.2025.1635425 doi:10.1007/s40618-024-02476-2 doi:10.3389/fonc.2022.975183 doi:10.3390/jimaging12040151 doi:10.1007/s00261-025-04893-0 doi:10.1007/s00330-023-10090-8	Quantifying subtle, visually imperceptible intratumoral heterogeneity, boundary irregularity, and vascularity variations associated with malignancy or endocrine activity.
Feature Selection & Dimensionality Reduction	LASSO regression, Minimum Redundancy Maximum Relevance (MRMR), ElasticNet, Pearson correlation analysis, Mutual Information (MI)	doi:10.1007/s00261-025-05345-5 doi:10.1186/s12880-023-01106-2 doi:10.1007/s00330-023-10090-8 doi:10.3892/ol.2024.14472 doi:10.3390/bioengineering10121423 doi:10.3389/fendo.2023.1265790 doi:10.3389/fonc.2025.1619341 doi:10.1016/j.acra.2025.12.026 doi:10.1186/s12880-026-02226-1	Identifying the most robust, non-redundant, and informative imaging biomarkers from high-dimensional datasets to effectively mitigate model overfitting.
Methodological Quality Assessment	Radiomics Quality Score (RQS), QUADAS-2 (Quality Assessment of Diagnostic Accuracy Studies 2)	doi:10.3389/fonc.2022.975183	Providing standardized evaluation frameworks to systematically audit the risk of bias, statistical power, and prospective translational readiness of radiomics models.

### 3.2 Applications in lesion differential diagnosis

Radiomics has demonstrated significant potential in improving the differential diagnosis of adrenal lesions, particularly in distinguishing benign adenomas from malignant tumors and other adrenal pathologies. Multiple studies have validated CT- and MRI-based radiomics models that outperform conventional imaging metrics such as unenhanced CT attenuation values alone. For instance, CT-based radiomics models have shown pooled sensitivities and specificities around 80% and 83%, respectively, with AUCs reaching 0.88 in differentiating malignant from benign adrenal tumors, indicating robust diagnostic performance (19). These models leverage texture and higher-order features that capture tumor heterogeneity and microstructural complexity beyond what is visually appreciable.

In the differentiation of adrenal adenomas from metastases, radiomics features reflecting intratumoral heterogeneity, such as entropy and non-uniformity, have been particularly informative. Studies have reported that malignant lesions and pheochromocytomas exhibit higher heterogeneity metrics compared to lipid-rich adenomas, enabling more accurate classification (22, 25). Clinical-imaging-radiomic nomograms combining radiomic scores with clinical factors such as age, sex, and CT attenuation values have further enhanced diagnostic accuracy, achieving AUCs above 0.9 in both training and validation cohorts (25). This integrative approach facilitates personalized risk stratification and may reduce unnecessary invasive procedures.

Radiomics also shows promise in distinguishing pheochromocytomas from other adrenal tumors, including lipid-poor adenomas and ACCs. Texture features indicative of necrosis, hemorrhage, and vascular patterns—such as gray-level zone emphasis and boundary low gray-level emphasis—have been identified as key discriminators (23, 26). Notably, interpretable artificial intelligence models using a minimal set of radiomic features have achieved accuracy rates exceeding 95% in differentiating pheochromocytomas from adenomas on contrast-enhanced CT, underscoring the clinical utility of radiomics in this context (23).

Moreover, radiomics aids in differentiating functioning adrenal adenomas, such as aldosterone-producing adenomas (APA), from NFA. Contrast-enhanced CT-based radiomics models combined with clinical data have demonstrated high diagnostic efficacy, with AUCs approaching 0.99 and sensitivities and specificities exceeding 90% (27). Similarly, MRI-based radiomics signatures have been developed to distinguish autonomous cortisol-secreting adenomas from non-functioning lesions, achieving an outstanding AUC of up to 0.99 in specific sequences (28), while demonstrating consistently promising diagnostic performance across external validation testing cohorts (29). These advances facilitate non-invasive functional assessment, potentially reducing reliance on biochemical testing.

In summary, radiomics enhances the differential diagnosis of adrenal lesions by quantifying imaging biomarkers related to tumor heterogeneity, vascularity, and microenvironmental changes. The integration of radiomic features with clinical and imaging parameters yields predictive models with superior accuracy compared to traditional imaging criteria. This progress supports more precise, non-invasive characterization of adrenal masses, guiding appropriate management and reducing unnecessary interventions.

### 3.3 Exploration in genotype prediction and prognostic assessment

Emerging evidence suggests that radiomics may extend beyond lesion characterization to the prediction of genetic alterations and prognostic outcomes in adrenal tumors. In patients with adrenal masses, advanced analytics are shifting from simple lesion characterization toward non-invasive germline and somatic genotype prediction. While preliminary studies in general AIs have explored germline genetic backgrounds and genotype–phenotype correlations in variants affecting genes such as ARMC5 or ZNRF3 (30), the most profound advancements in radiogenomic mapping have occurred in PPGLs. Integrated multi-platform molecular profiling has classified PPGLs into several molecularly defined subtypes, including pseudohypoxia, kinase signaling, Wnt-altered, and cortical admixture groups, each characterized by distinct genetic drivers and biological pathways (14).

The pseudohypoxia subtype is mainly associated with alterations involving SDHx, FH, VHL, and EPAS1, leading to aberrant hypoxia-inducible factor signaling, angiogenesis, metabolic reprogramming, and more aggressive biological behavior. Radiomically, this phenotype may manifest as pronounced intratumoral heterogeneity, irregular margins, and central cystic-necrotic degeneration, features that can be captured by higher-order texture matrices such as GLCM entropy and GLSZM non-uniformity (22). In contrast, the kinase signaling subtype is related to mutations in genes such as RET, NF1, HRAS, MAX, and TMEM127, which activate oncogenic pathways including MAPK and PI3K/AKT/mTOR. These tumors often present as well-circumscribed, predominantly solid, and hypervascular masses with relatively homogeneous enhancement patterns, which can be quantified using first-order attenuation features and shape descriptors such as roundness or sphericity (23).

The Wnt-altered subtype, driven by alterations such as MAML3 fusion or CSDE1 mutation, has been associated with adverse biological behavior, including metastatic potential (14). Radiomics and machine learning algorithms may capture subtle structural, surface-to-volume, and textural differences related to these molecular phenotypes and integrate them into risk classification frameworks such as the Grading system for Adrenal PPGLs (31). By leveraging multiphase CT or MRI radiomic descriptors, advanced classifiers may help non-invasively distinguish high-risk molecular subtypes from lower-risk tumors, thereby bridging macroscopic imaging phenotypes with underlying genomic and microenvironmental features (31). Although current data remain limited and largely exploratory, this approach may facilitate earlier identification of hereditary or biologically aggressive tumor syndromes and guide individualized management.

Regarding prognostic evaluation, radiomics has been applied to predict pathological grading and proliferative indices in ACC. Radiomic features extracted from CT and MRI have been linked to Ki-67 proliferation index and tumor aggressiveness, enabling risk stratification and individualized treatment planning (7, 19). For example, higher heterogeneity metrics and specific texture patterns correlate with more invasive tumor subtypes and poorer outcomes. Machine learning models incorporating radiomic signatures alongside clinical variables have demonstrated potential in predicting progression-free survival and guiding therapeutic decisions.

Furthermore, radiomics may assist in forecasting surgical complexity and outcomes. Studies employing radiomic features combined with clinical data have developed machine learning models to predict the difficulty of retroperitoneal laparoscopic adrenalectomy, aiding preoperative planning and risk mitigation (32). This application exemplifies the broader utility of radiomics in personalized patient management beyond diagnosis.

Despite these promising developments, translating radiomics for genotype prediction and prognostication into routine clinical practice remains restricted by retrospective designs and sample scarcity. The critical methodologies required to overcome these methodological heterogeneities and achieve multi-omics precision modeling are systematically appraised in subsequent sections of this review.

In conclusion, radiomics holds considerable promise for non-invasive prediction of genetic mutations and prognostic assessment in adrenal tumors. While preliminary findings are encouraging, further rigorous studies are required to establish validated imaging biomarkers that can reliably inform genetic risk and clinical outcomes, ultimately improving patient care.

## 4 The innovative role of deep learning in adrenal mass evaluation

### 4.1 Overview of deep learning models: from convolutional neural networks to transformers

CNNs have long been the cornerstone of DL architectures applied to medical imaging, including adrenal lesion assessment. CNNs leverage convolutional layers to automatically extract hierarchical features from images, progressing from low-level edge detection to high-level semantic representations. This hierarchical feature learning is critical in medical image analysis, where subtle texture and morphological variations can indicate different pathological states. The architecture typically includes convolutional layers for feature extraction, pooling layers for spatial dimension reduction, and fully connected layers for classification or regression tasks. In adrenal imaging, CNNs have been effectively utilized to differentiate between benign and malignant lesions, as well as to subtype adrenal adenomas based on CT or MRI data (15, 17). For instance, ResNet50-based CNN models have demonstrated high diagnostic accuracy in distinguishing lipid-poor adrenal adenomas from metastases, achieving AUC values exceeding 0.9 in internal and external validations (16).

Beyond classification, CNN variants such as U-Net and its derivatives have revolutionized medical image segmentation (21, 33). U-Net’s encoder-decoder structure with skip connections enables precise delineation of anatomical structures and lesions, which is essential for radiomics analysis where accurate lesion segmentation directly impacts feature extraction quality. In adrenal tumor evaluation, U-Net-based models facilitate automatic or semi-automatic segmentation of adrenal masses, significantly reducing the time and inter-observer variability associated with manual contouring. This segmentation step is a critical pre-processing stage for subsequent radiomics and DL analyses, enabling robust and reproducible feature extraction (34).

Recently, Transformer architectures, originally developed for natural language processing, have been adapted for vision tasks, including medical imaging. Vision Transformers (ViTs) employ self-attention mechanisms that capture long-range dependencies within images, overcoming the locality constraints of CNNs. This ability to model global context is particularly advantageous in complex imaging scenarios where lesion heterogeneity and surrounding tissue characteristics influence diagnosis. Emerging studies suggest that ViTs and hybrid CNN-Transformer models hold promise for adrenal lesion analysis, potentially enhancing feature representation and classification performance (35). Although still in early stages, these architectures may address limitations of CNNs in capturing spatial relationships across the entire image, offering a new frontier in adrenal imaging analysis.

In summary, the evolution from CNNs to Transformer-based models reflects a trajectory toward more sophisticated and context-aware image analysis techniques. CNNs remain the dominant architecture for adrenal imaging due to their proven efficacy and computational efficiency, especially in segmentation and classification tasks. However, the integration of Transformer models may further improve diagnostic accuracy by leveraging their global attention capabilities, heralding a new era in DL applications for adrenal tumor evaluation.

### 4.2 End-to-end diagnostic and classification applications

DL models have increasingly been employed in end-to-end diagnostic pipelines for adrenal lesion classification, bypassing traditional manual feature engineering. These models accept raw or minimally processed image inputs, such as ROI from CT or MRI scans, and directly output diagnostic predictions, including binary classifications (15, 17) (e.g., benign vs. malignant) or multi-class categorizations (e.g., adenoma subtypes, pheochromocytoma). This approach streamlines the workflow and reduces reliance on handcrafted radiomic features, which can be time-consuming and subject to variability.

Clinical studies have demonstrated that CNN-based models can achieve diagnostic performance comparable to, or even surpassing, that of experienced radiologists (15), particularly in challenging cases with atypical imaging features. For example, a ResNet50 CNN model combined with radiomics and clinical parameters achieved AUCs up to 0.996 in training cohorts for differentiating lipid-poor adrenal adenomas from metastases, with robust performance maintained in external validation sets (16). Similarly, DL models have shown high sensitivity and specificity in distinguishing ACC from large lipid-poor adrenal adenomas, successfully addressing a critical diagnostic challenge caused by overlapping imaging characteristics (17).

Moreover, multi-modal DL frameworks that integrate imaging data from multiple modalities—such as CT, MRI, and PET/CT—with clinical and biochemical parameters have shown promise in enhancing diagnostic accuracy and comprehensiveness (16). For instance, models incorporating 18F-Pentixafor PET/CT radiomics and DL features alongside clinical data successfully predicted CYP11B2-defined subtypes in primary aldosteronism, achieving an AUC of 0.907 and perfect sensitivity in test cohorts (36). Although the suitability of AI in nuclear medicine is sometimes debated—primarily because the high target-to-background ratios of PET tracers often yield visually definitive diagnoses that seemingly require no algorithmic assistance—AI proves to be highly valuable by transcending simple visual detection and maximum standardized uptake value (SUVmax) thresholding. AI is perfectly suited for nuclear medicine as it can quantify subtle spatial metabolic heterogeneity and facilitate multiparametric data fusion (10, 20). The integration of AI with specific advanced PET tracers significantly expands the horizons of adrenal imaging: (1) 18F-FDG PET and radiomics: This integration demonstrates exceptional accuracy in discriminating ACC and adrenal metastases from benign adenomas by capturing complex, sub-visual glycolytic distribution patterns (10, 20). (2) 68Ga-DOTA-SSA and 18F-FDOPA: Crucial for evaluating PPGLs, applying AI to these tracers (which target somatostatin receptors and catecholamine synthesis, respectively) can map intra-tumoral metabolic heterogeneity and potentially differentiate specific genetic clusters (e.g., SDHx versus VHL mutations) (10). (3) 68Ga-Pentixafor: Targeting CXCR4, its integration with AI and radiomics has proven highly effective in subtyping primary aldosteronism and predicting CYP11B2 expression, thereby offering non-invasive guidance for surgical decision-making (10, 36). (4) FAPI-PET: As an emerging modality, applying AI to fibroblast activation protein inhibitor (FAPI) PET could precisely quantify stromal activation and tumor microenvironment remodeling in aggressive adrenal malignancies like ACC, offering novel prognostic biomarkers that conventional imaging cannot provide. This integrative approach leverages complementary information from anatomical, functional, and molecular imaging, as well as patient-specific clinical context, enabling more precise and personalized diagnosis.

The ability of DL models to handle raw image data and fuse multi-modal inputs facilitates the development of clinically applicable tools that can assist radiologists and endocrinologists in decision-making. These models not only improve diagnostic accuracy but also reduce diagnostic time and inter-observer variability. The clinical translation of these end-to-end architectures relies on seamlessly conquering the inherent challenges of model opacity and generalizability, which are critically evaluated in the dedicated technological evolution frameworks below.

In conclusion, end-to-end DL applications represent a transformative advancement in adrenal lesion diagnosis, enabling automated, accurate, and comprehensive classification directly from imaging data. The integration of multi-modal data further augments diagnostic capability, paving the way for precision medicine approaches in adrenal tumor management.

### 4.3 Advantages of automated segmentation and feature learning

Automated segmentation driven by DL significantly enhances the efficiency and reproducibility of radiomics workflows in adrenal lesion evaluation. Manual delineation of adrenal tumors is labor-intensive, time-consuming, and subject to inter- and intra-observer variability, which can compromise the consistency of radiomic feature extraction and subsequent analyses. DL-based segmentation models, particularly those based on U-Net architectures (21, 33), enable rapid and precise delineation of adrenal masses, facilitating high-throughput and standardized radiomics studies.

The automation of segmentation not only expedites the workflow but also reduces subjective biases inherent in manual contouring. This improvement in reproducibility is crucial for the clinical translation of radiomics, where consistent and reliable feature extraction underpins diagnostic and prognostic modeling. For example, automatic segmentation combined with radiomics and clinical data has been shown to improve differentiation between lipid-poor adrenal adenomas and metastases, achieving AUCs up to 0.92 in combined models (34).

Beyond segmentation, DL models inherently learn hierarchical and complex features directly from imaging data, which may capture subtle and high-dimensional patterns beyond the scope of traditional handcrafted radiomic features. These learned features can reflect underlying pathophysiological processes more directly, offering novel imaging biomarkers for adrenal tumor characterization. Studies integrating DL signatures with radiomics (15, 16) have demonstrated superior diagnostic performance compared to radiomics or clinical models alone, suggesting that deep features provide complementary and biologically relevant information.

Furthermore, DL-based feature learning facilitates the discovery of new imaging markers that may correlate with molecular and genetic tumor characteristics (36, 37), potentially enabling non-invasive phenotyping and personalized treatment planning. For instance, models combining radiomics and DL features have been used to predict functional subtypes of adrenal adenomas, such as APA, correlating with hormonal secretion profiles and histopathological findings (36).

In summary, the advantages of automated segmentation and deep feature learning lie in enhanced workflow efficiency, improved reproducibility, and the potential to uncover novel, clinically relevant imaging biomarkers. These advances support the integration of DL into adrenal imaging pipelines, ultimately contributing to more accurate, objective, and personalized adrenal tumor assessment.

## 5 Integration strategies of radiomics and deep learning

### 5.1 Comparison and integration of “handcrafted features + machine learning” and “deep learning” approaches

The traditional “handcrafted features + machine learning” paradigm in radiomics has been widely applied in adrenal lesion evaluation due to its strong interpretability. This approach involves manual or semi-automatic extraction of predefined imaging features—such as shape, texture, intensity statistics, and wavelet transforms—from segmented adrenal lesions, which are then input into classical machine learning classifiers like random forests, support vector machines, or gradient boosting machines. The major advantage lies in the transparency of these features, which often have known clinical or biological correlates, facilitating clinical acceptance and hypothesis generation. For example, radiomic features reflecting lesion heterogeneity or texture have been linked to differentiating lipid-poor adrenal adenomas from metastases or pheochromocytomas, providing valuable diagnostic clues (18). However, this approach heavily depends on expert knowledge for feature engineering and selection, which may overlook complex, high-dimensional patterns embedded in imaging data. Moreover, handcrafted features may be sensitive to variations in image acquisition protocols and segmentation accuracy, limiting robustness and generalizability.

In contrast, DL models, particularly CNNs, automatically learn hierarchical feature representations directly from raw or minimally processed imaging data, obviating the need for manual feature design. This end-to-end learning capability enables DL to capture subtle and complex imaging patterns that may be imperceptible to human experts or traditional radiomics. Recent studies applying DL to adrenal mass differentiation have demonstrated promising diagnostic performance, such as distinguishing ACC from lipid-poor adenomas or differentiating pheochromocytoma from other adrenal lesions, often achieving high accuracy and sensitivity (15, 17). However, DL models are often criticized as “black boxes” due to their limited interpretability, which poses challenges for clinical trust and regulatory approval. Additionally, DL requires large, well-annotated datasets to avoid overfitting and ensure robustness, which can be difficult to obtain in adrenal tumor research given the relative rarity and heterogeneity of these lesions.

To leverage the complementary strengths of both approaches, fusion strategies have been proposed and validated. One common method involves using DL models for high-quality automatic segmentation of adrenal lesions, which then facilitates extraction of handcrafted radiomic features from precisely delineated volumes of interest, improving feature reliability and reproducibility (33, 34). Another integration strategy concatenates deep features learned by CNNs with handcrafted features, feeding the combined feature set into a downstream classifier such as XGBoost, which can enhance classification performance by capturing both explicit and implicit imaging characteristics (16). However, while such multi-model integrations achieve remarkable diagnostic accuracy, their clinical applicability remains low; the requirement for complex, computationally intensive preprocessing pipelines makes them difficult to deploy in standard, fast-paced clinical workflows.

To appreciate the paradigm shift brought by these artificial intelligence pathways, they must be rigorously contrasted against existing conventional diagnostic methods. Conventional evaluation relies on macro-morphological features, primarily the unenhanced CT density threshold of ≤10 HU to identify lipid-rich benign adenomas, supplemented by contrast washout kinetics or MRI chemical shift signal intensity indexes (2, 3, 11, 12). While these classic criteria are highly reliable for straightforward, lipid-rich masses, they lack structural granularity, scoring low in sensitivity when face-to-face with lipid-poor adenomas, atypical pheochromocytomas, or micro-metastases (3, 11). In comparison, the “Handcrafted Radiomics + Machine Learning” approach breaks medical images into high-dimensional quantitative data, utilizing advanced spatial matrices to capture subtle, sub-visual sub-tissue heterogeneity and microenvironmental complexity (5, 6). This quantitative granularity bridges the diagnostic “gray zone, “ though it introduces a dependency on manual segmentation and feature selection stability (6, 13). Moving further, “ DL “ models completely bypass human feature engineering, autonomously learning hierarchical, implicit pixel patterns directly from raw data arrays to classify indeterminate lesions or automatically segment boundaries with exceptional volumetric accuracy (15–17). While traditional methods excel in visual simplicity and instant clinical execution, they offer no capacity for prognostic stratification or molecular subtyping. Radiomics and DL sacrifice this immediate visual simplicity for unparalleled predictive depth, unlocking non-invasive forecasting of histopathological variants, treatment response, and genetic mutations that conventional imaging cannot discern (7, 8, 36).

Furthermore, explainable artificial intelligence (XAI) techniques have been increasingly integrated to dismantle the “black box” nature of these networks. While feature-based machine learning and hybrid networks primarily leverage SHAP to quantify global and local feature importance (15, 34), end-to-end DL architectures also utilize pixel-level attribution methods such as Gradient-weighted Class Activation Mapping (Grad-CAM) and Local Interpretable Model-agnostic Explanations (LIME) to visualize salient diagnostic regions directly on CT or MRI slices, thereby increasing model transparency and clinical acceptability. For instance, SHAP analysis has elucidated the contribution of clinical and imaging features alongside DL outputs in differentiating lipid-poor adenomas from metastases or pheochromocytomas, guiding clinicians in understanding model rationale.

**Figure 2:**
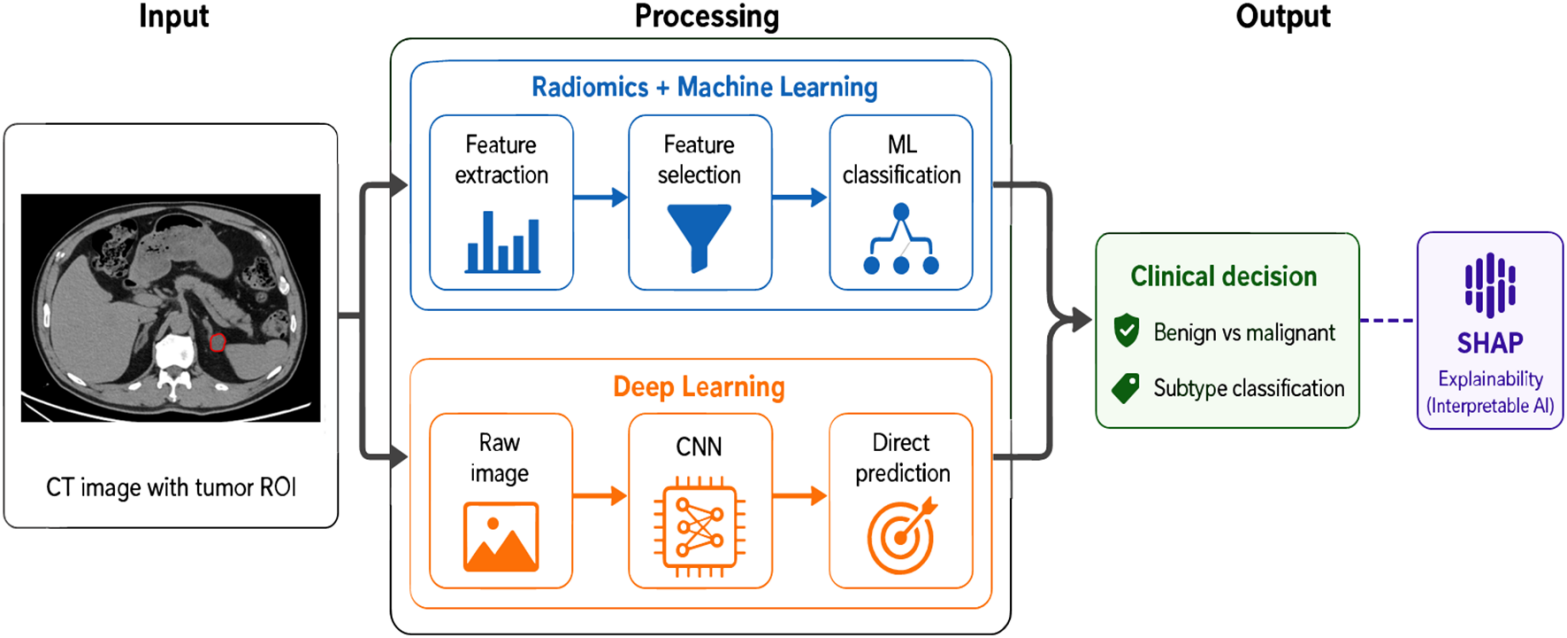
Radiomics- and deep learning-based analytical pathways for adrenal mass evaluation. The schematic illustrates two AI-based workflows starting from a CT image with a tumor region of interest. The radiomics pathway includes feature extraction, feature selection, and machine learning classification, whereas the deep learning pathway directly processes image data using convolutional neural networks. The outputs support clinical decision-making, including benign–malignant differentiation and subtype classification, with SHAP providing model interpretability.

In summary, while handcrafted feature-based machine learning offers interpretability and clinical insight, it may miss complex patterns, whereas DL excels in feature learning but suffers from opacity and data demands. Their integration through segmentation-assisted radiomics, feature fusion, and XAI represents a promising avenue to harness the advantages of both, improving diagnostic accuracy and fostering clinical translation in adrenal mass evaluation. The distinct technical workflows of these two standard artificial intelligence pathways, spanning from initial image input through feature processing to the final explainable clinical decision, are schematically illustrated in **Figure 2**. To provide a structured and comprehensive overview of these methodologies in clinical scenarios, **Table 2** systematically summarizes representative radiomics and DL studies, highlighting their diagnostic tasks, imaging modalities, cohorts, analytical frameworks, and key performance metrics.

**Table 2:** Summary of representative radiomics and deep learning studies in adrenal mass evaluation.

Reference	Diagnostic / clinical task	Modality & cohort size (n)	Segmentation & modeling methodology	Key performance & results	Key limitations & clinical applicability
doi:10.1186/s12880-025-01842-7	Differentiating large (≥ 4 cm) ACC from PHEO	Contrast-enhanced CT; n = 158 (Multicenter)	Manual 3D segmentation; Handcrafted radiomics + SHAP interpretability	External test accuracy: 86%, Specificity: 88%, Sensitivity: 81% (AUC: 0.920)	Moderate Applicability: Has external validation and SHAP, but manual segmentation limits high-throughput clinical use.
doi:10.1007/s00261-025-05345-5	Differentiating lipid-poor adrenal adenoma from metastasis	Contrast-enhanced CT; n = 535 (Multicenter)	Fully automated segmentation; ResNet50 deep features + Radiomics + SHAP	Combined model AUC: 0.996 (Training), 0.939 (Internal Val), 0.852 (External Test)	Moderate Applicability: Robust validation, but the highly complex dual-extraction pipeline is difficult to integrate into standard PACS.
doi:10.1007/s00261-023-03988-w	Distinguishing stage 1–2 ACC from large lipid-poor adenoma	Single time-point CT; n = 91	Annotated single-slice ROI; 3D DenseNet121 architecture	Accuracyfocused model: 91% accuracy, 96% sensitivity; Sensitivityfocused: 87.2% accuracy, 100% sensitivity	Low Applicability: Small single-center cohort with no external validation; high risk of model overfitting.
doi:10.1186/s12880-023-01106-2	Differentiating lipid-poor adenoma from subclinical PHEO	Multiphase spiral CT; n = 134 (Multicenter validation)	3D Slicer manual segmentation; PyRadiomics + 6 machine learning classifiers (MLP, SVM, etc.)	Non-contrast test set AUC: 0.919–0.981; Multi-layer Perceptron (MLP) achieved optimal validation AUC (0.979)	High Applicability: Relies only on non-contrast CT, simplifying workflow, though manual segmentation remains a bottleneck.
doi:10.1007/s00261-025-04893-0	Differentiating adrenal pheochromocytoma from adenoma	Contrast-enhanced CT; n = 152	Confirmed automated segmentation; 463 radiomic features + Rule-learning interpretable AI	3-feature optimal rule achieved F1-score of 0.97 (Training) and 0.96 (Test set)	Moderate Applicability: Highly interpretable rules, but lacks multicenter external validation to confirm the rules aren't overfitted to local data.
doi:10.1186/s12880-025-01835-6	Distinguishing PHEO from large-diameter lipid-poor adenoma	Multiphase CT; n = 202 (Multicenter)	Manual segmentation; Combined Clinico-radiological-radiomics Nomogram	Nomogram AUC: 0.994 (Training), 0.979 (Validation), 0.945 (External Test)	Moderate Applicability: Good multicenter validation, but heavily dependent on multi-phase scanning which increases radiation dose.
doi:10.3390/bioengineering10121423	Differentiating aldosterone-producing adenoma (APA) from NAA	Contrast-enhanced CT; n = 128	Univariate + LASSO selection; Logistic Regression Machine Learning Classifier	Radscore-only AUC: 0.869; Integrated Clinic-Radscore combined model AUC: 0.994	Low Applicability: No external validation cohort; very high AUC on a small sample suggests a high risk of overfitting.
doi:10.5114/pjr.2023.124435	Determining adrenal Cushing's syndrome (ACS) vs. NFAI	3-Tesla MRI (In-phase, Opposed-phase, T2W); n = 50	3D lesion manual segmentation; LASSO feature regression + Radiomic scoring	T1W opposed-phase AUC: 0.862; T1W in-phase AUC: 0.892; T2W radiomic signature AUC: 0.994	Low Applicability: Extremely small sample size (n=50) and lack of external testing indicate a severe risk of overfitting.
doi:10.3389/fendo.2023.1265790	Predicting surgical difficulty of retroperitoneal laparoscopic adrenalectomy	NECT & CECT; n = 300+ (Retrospective + Prospective)	3D Slicer manual segmentation; LASSO feature selection + RF / XGBoost + SHAP	Random Forest (RF) demonstrated stable performance; Rad-score exerted the highest predictive impact	Moderate Applicability: Strong prospective validation included, but limited to a single center's surgical expertise and imaging protocols.
doi:10.1016/j.acra.2025.12.026	Non-invasive prediction of CYP11B2-defined PA pathological subtypes	18F-Pentixafor PET/CT; n = 89	Stepwise regression + MRMR feature selection; Support Vector Machine (SVM) + SHAP	Combined multi-modal model outperformed single feature inputs, achieving Test set AUC: 0.907, F1-score: 0.923	Low Applicability: Small cohort size risks overfitting; furthermore, 18F-Pentixafor PET/CT is expensive and not universally available.
doi:10.1186/s12880-026-02226-1	Preoperative prediction of aldosterone secretion and CYP11B2 status	Contrast-enhanced CT; n = 140 (External validation)	ElasticNet feature selection; Combined Clinic-radiomics integration + SHAP evaluation	Combined model achieved average AUC values of 0.912, 0.923, and 0.958 for distinct histopathological subtyping	Moderate Applicability: Includes external validation, but the retrospective nature requires prospective trials before clinical adoption.

### 5.2 Construction of multicenter studies and standardized platforms

The clinical translation of radiomics and DL models for adrenal lesion assessment critically depends on their generalizability and robustness across diverse patient populations and imaging settings. Achieving this requires large-scale, multicenter studies that aggregate heterogeneous datasets encompassing variations in scanner types, imaging protocols, and patient demographics. Such multicenter collaborations enable comprehensive model training and validation, mitigating overfitting to single-center biases and enhancing external validity. For example, recent studies involving hundreds of patients from multiple tertiary centers have demonstrated improved model performance and adaptability in differentiating adrenal adenomas from metastases or pheochromocytomas by leveraging diverse imaging data (16, 34). However, assembling such datasets poses challenges related to data privacy, heterogeneity, and standardization.

To address inter-center variability, establishing unified imaging acquisition protocols is essential. Standardization of parameters such as slice thickness, radiation dose, contrast phases, and reconstruction algorithms reduces technical heterogeneity that can adversely affect radiomic feature stability and DL model performance. Preprocessing pipelines including image normalization, resampling, and artifact correction should also be harmonized to ensure consistent input quality. For instance, studies have shown that models trained on data with standardized protocols exhibit higher reproducibility and diagnostic accuracy compared to those trained on heterogeneous datasets (33). Moreover, consensus guidelines and quality control frameworks for adrenal imaging can facilitate protocol harmonization across institutions.

Emerging technological solutions further enable collaborative model development while preserving patient privacy. Cloud-based platforms provide centralized environments for data storage, annotation, and model training accessible to multiple centers, fostering efficient data sharing and joint analysis. More importantly, federated learning techniques allow decentralized training of machine learning models on local datasets without transferring raw data, thus complying with privacy regulations such as GDPR and HIPAA. This approach aggregates model updates rather than patient data, enabling robust model development across institutions while safeguarding confidentiality (38). Federated learning has shown promise in other oncologic imaging domains and is poised to accelerate adrenal imaging AI research by overcoming data-sharing barriers.

In conclusion, the construction of large-scale, multicenter, and standardized imaging databases is indispensable for developing generalizable radiomics and DL models in adrenal mass evaluation. Harmonized imaging protocols, standardized preprocessing, and privacy-preserving collaborative platforms such as cloud computing and federated learning constitute the foundational infrastructure to realize the clinical potential of AI-driven adrenal imaging diagnostics.

## 6 Current challenges and limitations

### 6.1 Technical methodological challenges

The application of radiomics and DL in adrenal mass evaluation faces significant technical methodological challenges that hinder their widespread clinical adoption. A primary concern is the stability and reproducibility of radiomic features, which are highly sensitive to variations in imaging acquisition parameters, scanner types, reconstruction algorithms, and ROI segmentation methods. For instance, studies have demonstrated that radiomic features extracted from contrast-enhanced CT or MRI images can vary substantially depending on the scanner vendor and imaging protocols, leading to inconsistent feature values and reduced reliability across centers (13, 34). This lack of standardization complicates the development of robust predictive models and limits their generalizability.

Moreover, the segmentation of adrenal lesions, whether manual, semi-automatic, or fully automatic, introduces variability that further impacts feature extraction. Although automatic segmentation techniques have been developed to improve efficiency and consistency, their accuracy can be compromised by lesion heterogeneity, size, and imaging artifacts, especially in lipid-poor adenomas or atypical masses (16, 36).

Another critical challenge is model overfitting and limited generalizability. Many existing studies rely on single-center datasets with relatively small sample sizes, which increases the risk of overfitting where models perform well on training data but poorly on external validation cohorts. For example, while some DL models differentiating ACC from lipid-poor adenomas report high internal accuracy, their reliance on small, single-center cohorts without external validation inherently carries a high risk of overfitting, resulting in low immediate clinical applicability (17). The scarcity of large, diverse datasets is particularly problematic for rare adrenal tumors such as ACC or pheochromocytoma, where the “small data” problem restricts the ability to train DL models with sufficient statistical power and diversity. This limitation is compounded by the difficulty in obtaining high-quality, pathologically confirmed labels and comprehensive clinical annotations necessary for supervised learning (16, 17).

Furthermore, the heterogeneity of adrenal lesions, including variations in size, lipid content, and functional status, adds complexity to model development. Radiomics and DL models must capture subtle imaging differences that distinguish benign from malignant or functioning from non-functioning lesions, which is challenging given overlapping imaging characteristics and the presence of atypical or mixed tumors (11, 18). Finally, the integration of multimodal data—combining radiomics, DL features, and clinical parameters—has shown improved diagnostic performance but also increases model complexity and the risk of overfitting if not carefully managed with appropriate feature selection and validation strategies (15, 16).

In summary, overcoming these technical challenges requires standardized imaging protocols, robust and reproducible feature extraction methods, larger multicenter datasets for training and validation, and advanced model development techniques that mitigate overfitting while enhancing generalizability. Only then can radiomics and DL achieve reliable and reproducible performance in adrenal mass evaluation.

### 6.2 Clinical translation and integration barriers

Despite promising advances in radiomics and DL for adrenal mass assessment, significant barriers remain in translating these technologies into routine clinical practice. A major obstacle is the paucity of prospective, multicenter clinical validation studies that rigorously evaluate the incremental clinical value of these AI models over existing diagnostic standards. Most current evidence derives from retrospective single-center cohorts, limiting the demonstration of real-world utility and generalizability across diverse patient populations and imaging platforms (16, 17). Without robust prospective validation, clinicians remain cautious about adopting AI tools that have not been proven to improve diagnostic accuracy, patient outcomes, or cost-effectiveness in routine workflows.

Another critical challenge is the limited interpretability and explainability of AI models, particularly DL networks, which are often perceived as “black boxes.” This opacity hinders clinicians’ trust and acceptance, especially among non-radiology specialists who may be unfamiliar with AI methodologies. Efforts to enhance model transparency using techniques such as SHAP have been applied in adrenal tumor classification to elucidate feature contributions and improve interpretability, yet these remain underutilized in clinical settings (15, 16). Improving explainability is essential to facilitate clinician understanding, enable informed decision-making, and support regulatory approval.

Furthermore, integrating AI model outputs seamlessly into existing clinical workflows and decision support systems presents technical and operational challenges. Current clinical environments rely on established imaging platforms, electronic health records (EHRs), and multidisciplinary tumor boards, which may not be readily compatible with AI tools requiring additional software, data preprocessing, or manual intervention. Efficient integration demands interoperable systems that can automatically process imaging data, generate AI predictions, and present results in user-friendly formats without disrupting clinical routines (26, 34).

Additionally, workflow integration must address data privacy, security, and compliance with healthcare regulations. The lack of standardized protocols for AI deployment, including quality control, model updating, and performance monitoring, further complicates clinical adoption. Finally, clinician education and training are vital to bridge the gap between AI technology and clinical practice. Physicians need to understand the capabilities, limitations, and appropriate use cases of AI models to effectively incorporate them into patient management. Multidisciplinary collaboration among radiologists, endocrinologists, surgeons, and data scientists is necessary to develop clinically relevant AI solutions and foster acceptance (39, 40).

In conclusion, overcoming clinical translation and integration barriers requires prospective multicenter validation, enhanced model interpretability, seamless workflow integration, regulatory compliance, and clinician engagement. Addressing these challenges will be pivotal to realizing the full potential of radiomics and DL in improving adrenal mass evaluation and patient care. To bridge the gap between current bottlenecks and subsequent clinical deployment, **Table 3** provides a structured mapping of these technical and clinical barriers alongside their prospective mitigating strategies and technological solutions.

**Table 3:** Mapping of current methodological/clinical challenges to future technological solutions.

Dimension	Core challenge & current limitation	Future strategy & technological solution	Representative references
Technical & Reproducibility	High sensitivity of radiomic features to imaging protocols, reconstruction variations, and scanner vendors across institutions.	Harmonized preprocessing pipelines (voxel resampling, intensity normalization) and adherence to universal imaging standards.	doi:10.2174/0115734056399745260330003149 doi:10.3389/fonc.2022.975183 doi:10.1177/17562872251352553 doi:10.3390/life15040560 doi:10.3389/fonc.2025.1619341 doi:10.1007/s10278-024-01158-y
Data Scarcity & Heterogeneity	The "small data" restriction due to the rarity of specific aggressive subtypes (e.g., ACC, malignant PPGL), leading to overfitting risks.	Implementation of advanced learning paradigms: small-sample learning, transfer learning, and GAN-based synthetic data augmentation.	doi:10.2174/0115734056399745260330003149 doi:10.1007/s00261-025-05345-5 doi:10.1007/s00261-023-03988-w
Model Interpretability	The opaque "black box" nature of deep learning networks, which hinders clinical trust and regulatory approvals.	Adoption of a comprehensive multi-method XAI paradigm (integrating SHAP, Grad-CAM, LIME, Integrated Gradients, attention maps, and occlusion analysis) tailored to both hybrid radiomics and end-to-end vision networks.	doi:10.1186/s12880-025-01842-7 doi:10.1186/s12880-025-01798-8 doi:10.1007/s00261-025-05345-5 doi:10.3389/fonc.2025.1619341 doi:10.1016/j.acra.2025.12.026 doi:10.1186/s12880-026-02226-1
Evidence & Generalizability	Predominance of retrospective, single-center study designs lacking prospective external validation in diverse real-world cohorts.	Designing prospective multicenter clinical trials and employing privacy-preserving Federated Learning architectures for decentralized training.	doi:10.1186/s12880-025-01798-8 doi:10.1007/s00261-025-05345-5 doi:10.1007/s00261-023-03988-w doi:10.3389/fonc.2022.975183 doi:10.1177/17562872251352553 doi:10.3389/fonc.2025.1619341
Workflow Integration	Operational incompatibility with established healthcare IT ecosystems (PACS, EHR) and multidisciplinary clinical routines.	Development of standardized, automated end-to-end user software and decision support interfaces integrated directly into clinical platforms.	doi:10.1007/s00261-025-05345-5 doi:10.1186/s12880-025-01835-6 doi:10.3389/fonc.2025.1619341 doi:10.3803/EnM.2023.1779 doi:10.1210/endrev/bnaa008

### 6.3 Ethical considerations, data privacy, and algorithmic bias

As artificial intelligence transitions from retrospective research pipelines into active clinical decision support systems for adrenal oncology, addressing ethical implications, data privacy safeguards, and algorithmic bias becomes paramount.

First, data privacy remains a fundamental challenge due to the massive volume of high-resolution digital imaging data (CT/MRI/PET) and associated clinical-biochemical records required to train reliable neural networks. To comply with strict international legal frameworks such as the General Data Protection Regulation (GDPR) and the Health Insurance Portability and Accountability Act (HIPAA), data-sharing across medical centers demands rigorous, multi-tier de-identification protocols (16, 38). Standard removal of metadata tags is frequently insufficient; emerging concerns regarding the theoretical re-identification of patients through unique anatomical reconstructions of the spine or surrounding musculature on large abdominal fields of view necessitate advanced cryptographic solutions like federated learning architectures, which perform decentralized model training without moving raw patient data from local institutional silos (38).

Second, algorithmic bias represents a significant barrier to equity in healthcare delivery. Machine learning models are highly susceptible to data source biases; a network trained exclusively on data from a single tertiary reference center using scanners from a single manufacturer will inherently develop a performance bias skewed toward those specific technical parameters (16, 19). When deployed externally, such models often experience sharp declines in diagnostic accuracy due to variations in reconstruction kernels, slice thickness, or radiation doses across different hospitals (19, 41).

Furthermore, demographic bias arises when the underlying training cohorts fail to reflect ethnically or geographically diverse populations, potentially leading to misclassifications in rare conditions like ACC. Ensuring ethical deployment requires developers to actively implement algorithmic fairness audits, diversify training distributions, and establish transparent data-provenance baselines to mitigate the compounding of clinical disparities through automated software.

## 7 Future prospects and development directions

### 7.1 Technological evolution: toward greater precision and interpretability

The evolution of imaging and computational technologies has been pivotal in advancing the precision and reliability of adrenal mass evaluation through radiomics and DL. A critical technical challenge lies in developing radiomic features and preprocessing pipelines that are robust to variations in image acquisition parameters, such as differences in CT scanner models, contrast phases, and imaging protocols. Studies have demonstrated that standardizing preprocessing workflows—including normalization, resampling, and segmentation protocols—can significantly enhance feature reproducibility and model stability across multicenter datasets (41). This standardization is essential to mitigate the heterogeneity inherent in clinical imaging data and to ensure that extracted features truly reflect underlying tissue characteristics rather than technical artifacts (19, 20).

Addressing the scarcity of data, especially for rare adrenal tumors like PPGLs, has driven the exploration of advanced machine learning strategies such as small-sample learning, transfer learning, and generative adversarial networks (GANs) for data augmentation. These approaches enable models to leverage knowledge from related domains or synthetically expand datasets, thereby improving generalizability and reducing overfitting risks (13). For instance, GAN-based augmentation has been used to enhance the diversity of training samples, facilitating more robust classification of lipid-poor adenomas versus metastases (42). Transfer learning from large-scale imaging datasets further accelerates model convergence and performance, particularly when labeled adrenal tumor data are limited.

Parallel to these developments, the field has witnessed a surge in the integration of XAI techniques to enhance model transparency and foster clinician trust. Methods such as attention maps, feature inversion, and SHAP have been employed to elucidate the contribution of specific radiomic features or image regions to model predictions. For example, SHAP analysis has been instrumental in interpreting complex models differentiating adrenal surgical difficulty or malignant tumor lineages (9, 15, 32), revealing that radiomic signatures often dominate predictive importance alongside key clinical variables. To build a truly robust framework for clinical trust, the future technological trajectory must move beyond an exclusive reliance on SHAP toward a comprehensive multi-method XAI paradigm that encompasses *post-hoc* local attributions, perturbation-based analysis, and intrinsic interpretable mechanisms. Specifically, the integration of these distinct XAI methodologies offers complementary insights into adrenal image analysis: (1) Grad-CAM: As a gradient-based localization technique, it computes the gradients of the score for a specific class with respect to the final convolutional layer activations, producing coarse localization maps that highlight the exact discriminatory regions (such as focal necrosis, vascular patterns, or irregular borders) within an indeterminate adrenal mass. (2) Integrated Gradients: This approach satisfies the fundamental axioms of sensitivity and implementation invariance by integrating gradients along a straight path from a baseline blank image to the input adrenal scan, yielding pixel-level attribution maps with much sharper fidelity than standard visual saliency methods. (3) LIME: By training local, interpretable surrogate models (such as sparse linear regressors) around individual adrenal lesion predictions, LIME explains specific anomalous classifications in a model-agnostic manner. (4) Attention Maps: Intrinsically embedded within emerging ViTs, attention maps visualize the self-attention weights across image patches, elucidating how global context and long-range spatial relationships between the adrenal gland and surrounding retroperitoneal structures influence the final diagnostic output (35). (5) Occlusion Analysis: This perturbation-based method systematically masks different patches of the adrenal ROI with a grey box and measures the corresponding drop in classification probability, providing a highly intuitive and model-agnostic verification of feature importance. Synthesizing these diverse explainability tools will allow radiologists to cross-verify algorithmic outputs, ensuring that convolutional networks are making decisions based on genuine pathophysiological imaging biomarkers rather than confounding technical artifacts. This interpretability not only aids in clinical acceptance but also provides insights into tumor biology, potentially uncovering novel imaging biomarkers. The establishment of “human-machine trust” through such transparent AI systems is a crucial step toward clinical translation, ensuring that automated decisions can be audited and understood by healthcare professionals.

In summary, the technological trajectory in adrenal imaging radiomics and DL is characterized by efforts to enhance feature robustness against imaging variability, innovative solutions to data scarcity via advanced learning paradigms, and the critical incorporation of explainability frameworks. These advances collectively push the field toward more precise, reliable, and clinically interpretable models, laying the groundwork for their integration into routine adrenal mass assessment.

### 7.2 Expansion of clinical applications: beyond diagnosis

While initial applications of radiomics and DL in adrenal imaging have focused predominantly on differential diagnosis—distinguishing benign from malignant lesions or subtyping adenomas—the clinical utility of these technologies is rapidly expanding into comprehensive disease management. Current research is extending the scope from static diagnosis to dynamic prediction of therapeutic responses, longitudinal efficacy evaluation, and recurrence monitoring, thereby supporting full-cycle patient care. For example, radiomics models have been developed to predict response to chemotherapy in adrenal tumors, enabling personalized treatment planning and early identification of non-responders (8). Such predictive capabilities are crucial in tailoring interventions and optimizing outcomes in heterogeneous adrenal neoplasms.

A major frontier is the deepening of radiogenomic correlations, which aim to bridge imaging phenotypes with underlying molecular and genetic alterations. By integrating radiomic features with histopathological and immunohistochemical data, studies have begun to establish reliable non-invasive subtyping of adrenal tumors, such as linking specific radiomic signatures to protein expression profiles (e.g., CYP11B2 status) implicated in tumorigenesis (37). This approach promises to obviate the need for invasive biopsies, facilitating precision oncology through imaging biomarkers that reflect tumor biology at the molecular level. For instance, PET/CT radiomics combined with machine learning has been shown to predict CYP11B2 expression in primary aldosteronism, a key molecular marker, thereby guiding subtype-specific management (36).

Moreover, the potential of radiomics and DL features as imaging biomarkers is being explored in clinical trials for patient stratification and endpoint evaluation. These quantitative imaging markers can serve as surrogate endpoints, enabling more sensitive and earlier assessment of treatment efficacy compared to conventional imaging criteria. For example, radiomics-based risk stratification models for pheochromocytoma have been proposed to select patients for clinical trials targeting high-risk phenotypes (31). The integration of such biomarkers into trial design enhances the precision of patient selection and the robustness of outcome measures, accelerating the development of novel therapeutics.

**Figure 3:**
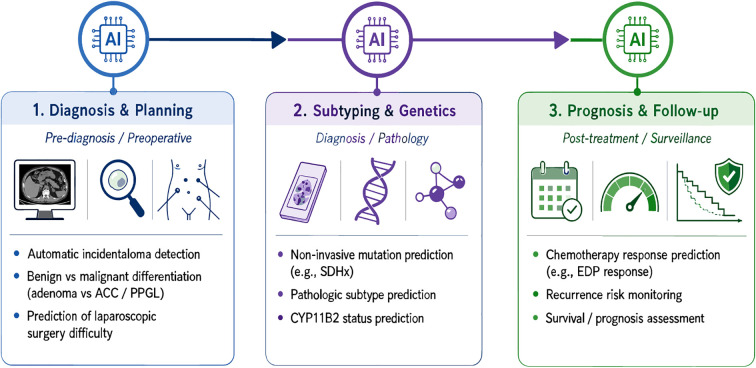
Clinical application timeline of AI in adrenal mass evaluation. This schematic summarizes the potential applications of radiomics and deep learning across the clinical course of adrenal mass management. AI may support diagnosis and preoperative planning, assist non-invasive subtyping and genetic or molecular prediction, and contribute to post-treatment surveillance, including treatment response prediction, recurrence monitoring, and prognostic assessment.

Collectively, these advances signify a paradigm shift from diagnostic imaging toward a holistic, image-guided precision medicine framework in adrenal tumor management. Radiomics and DL are evolving into indispensable tools for predictive analytics, molecular characterization, and therapeutic monitoring, thereby extending their impact well beyond initial diagnosis. The clinical application timeline of these artificial intelligence tools across the comprehensive patient journey, transitioning from initial diagnosis and surgical planning to pathological subtyping and longitudinal prognosis, is schematically illustrated in **Figure 3**.

### 7.3 Toward clinical practice and personalized medicine

The translation of radiomics and DL technologies from research to routine clinical practice in adrenal mass evaluation necessitates rigorous validation and user-centric implementation. A critical step is the design and execution of prospective, multicenter clinical trials with standardized imaging protocols and robust endpoints to generate high-level evidence supporting clinical utility. Such trials are essential to confirm diagnostic accuracy, prognostic value, and impact on patient outcomes across diverse populations and healthcare settings (19). They also facilitate regulatory approval and guideline incorporation, which are prerequisites for widespread adoption (39).

Parallel to evidence generation, the development of user-friendly software platforms and integrated clinical decision support systems is imperative. These tools must comply with clinical workflow requirements, including seamless integration with picture archiving and communication systems (PACS), EHRs, and standardized reporting formats. Simplifying the user interface and automating complex processes such as lesion segmentation, feature extraction, and model inference reduce barriers to adoption among radiologists and clinicians. For example, automatic segmentation combined with radiomics and DL models has demonstrated high diagnostic performance in differentiating lipid-poor adenomas from metastases, highlighting the feasibility of fully automated pipelines (16, 34).

**Figure 4:**
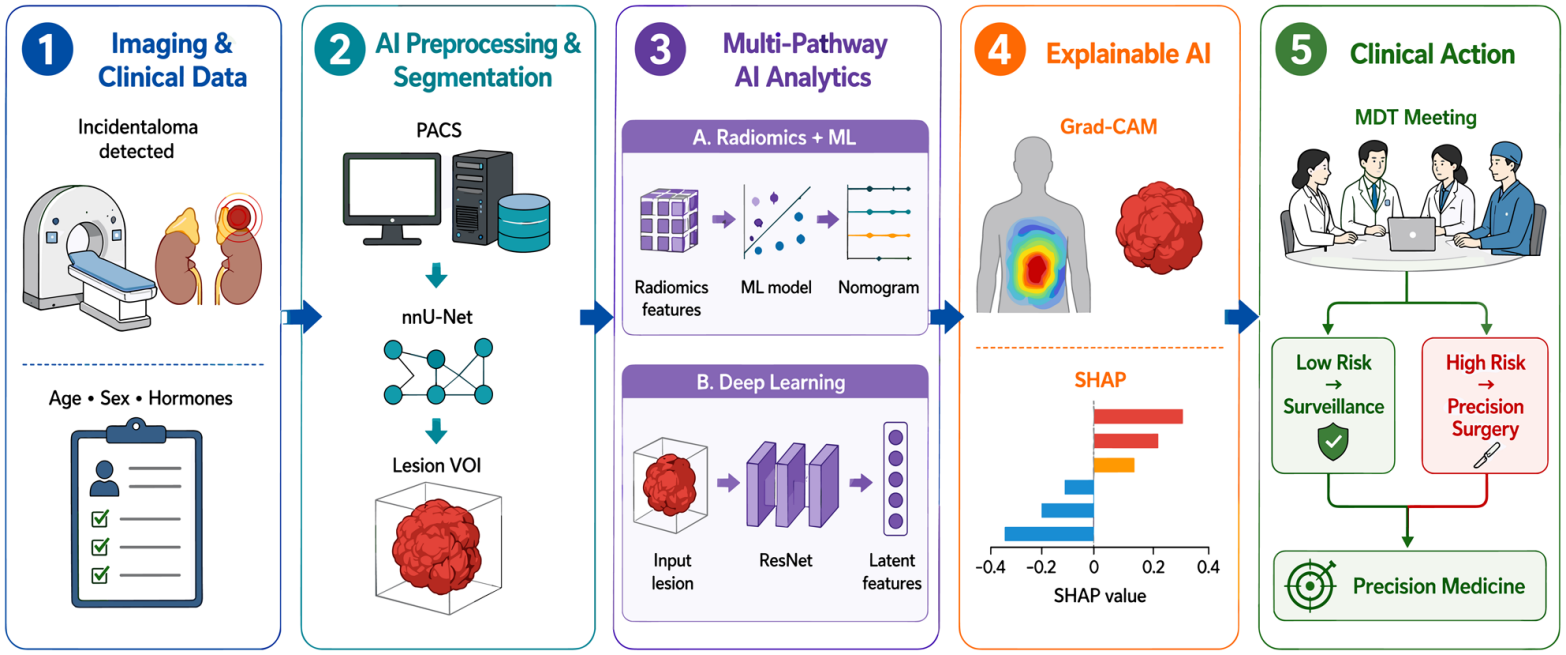
Simplified AI-assisted clinical workflow for adrenal mass evaluation. This schematic illustrates a five-step workflow for AI-assisted adrenal mass evaluation, including data acquisition, AI preprocessing and segmentation, parallel radiomics/machine learning and deep learning analysis, explainable AI interpretation, and multidisciplinary clinical decision-making for personalized management.

The ultimate objective is to establish intelligent, comprehensive decision support systems that integrate automated detection, segmentation, diagnosis, and prognostic prediction into a unified platform. Such systems would provide personalized risk assessments and management recommendations for adrenal masses, supporting precision medicine approaches. The integrated operational architecture of this end-to-end framework, encompassing everything from initial patient image acquisition to downstream AI-driven decision support, is schematically mapped in **Figure 4**. For instance, combined clinical-radiomic models predicting the difficulty of retroperitoneal laparoscopic adrenalectomy enable tailored preoperative planning, potentially reducing operative risks and improving outcomes (32).

To successfully bridge the gap between algorithmic capability and real-world utility, it is critical to define the exact clinical implications and specific patient cohorts where AI can disrupt standard care pathways.

First, under what circumstances can AI change current clinical guidelines: Current international guidelines, such as the 2023 European Society of Endocrinology (ESE) standards, rely heavily on a rigid morphological threshold—specifically, an unenhanced CT attenuation value of ≤10 HU—to definitively classify a unilateral adrenal mass as benign (2, 3, 39, 40). AI can formally change these guidelines only when large-scale, prospective, multicenter trials achieve a validated negative predictive value (NPV) approaching 100% for identifying benignity in lesions exceeding 10 HU. Once AI models can reliably replicate this safety margin across diverse scanner vendors, guidelines can safely evolve to substitute or complement the static 10 HU rule with multi-parametric radiomic signatures.

Second, in which patients can AI reduce unnecessary adrenalectomies: This intervention is most impactful for patients presenting with large (≥4 cm) yet indeterminate lipid-poor adrenal adenomas or non-functioning lesions. Under current protocols, large-diameter or hyperattenuating incidentalomas frequently prompt surgical resection due to overlapping morphological features with early-stage ACC or localized metastases (17). By deploying validated CNNs and handcrafted radiomics capable of detecting deep microstructural homogeneity, clinicians can confidently manage these large, morphologically suspicious but biologically benign adenomas conservatively, thereby sparing patients from the risks of unnecessary adrenalectomies (16, 17, 34).

Third, in which patients can AI reduce the need for biochemical testing: This directly applies to patients with incidentally discovered, indeterminate lipid-poor masses where differentiating NFA from subclinical, biochemically silent pheochromocytomas or autonomous hormone-secreting tumors is highly complex. Currently, all such patients must undergo exhaustive, time-consuming endocrine workups, including dexamethasone suppression and metanephrine testing. Advanced machine learning classifiers trained on unenhanced or dynamic contrast CT radiomics have demonstrated outstanding efficacy in non-invasively ruling out functional secretory activity—such as robustly separating LPA from sPHEO, APA from non-functioning lesions, and autonomous cortisol-secreting tumors from NFA (18, 27–29). In patients classified by AI as having an extremely low probability of hormonal activity, the immediate necessity for exhaustive multi-tier biochemical testing could be safely reduced or streamlined (18).

Fourth, in which patients can AI change the indication for PET: 18F-FDG PET/CT is routinely indicated to differentiate primary malignant ACC or metastatic disease from atypical benign masses when conventional washout CT or MRI yields equivocal results. AI can alter this indication by serving as an effective non-invasive gatekeeper for lung cancer or other oncological patients who present with concurrent small, hyperattenuating AIs (25). Integrated clinical-radiological-radiomic nomograms based on baseline plain or contrast CT have shown high diagnostic performance that matches or exceeds visual interpretation by radiologists. For patients whom the CT-based AI nomogram classifies as having an indisputably benign profile with a high NPV, the clinical indication to perform a confirmatory 18F-FDG PET/CT scan can be safely bypassed, significantly reducing healthcare costs and patient radiation exposure.

The integration of multi-omics data, real-time intraoperative guidance, and continuous learning capabilities represent future directions to enhance system intelligence and clinical relevance.

In conclusion, advancing radiomics and DL into clinical practice requires a concerted effort encompassing rigorous validation, development of accessible and standardized tools, and construction of intelligent decision support systems. These endeavors will facilitate personalized, precise, and efficient management of adrenal masses, ultimately improving patient care and outcomes. To practicalize this future vision, the scientific community must actively address three immediate research gaps and prioritize the following areas for future investigation: (1) Prospective Multicenter Trial Verification: Current literature is overwhelmingly dominated by retrospective, single-center study designs; there is a critical knowledge gap regarding how these AI tools perform dynamically in real-world clinical environments. Future work must prioritize large-scale prospective trials to confirm that AI recommendations safely improve patient outcomes and save medical costs. (2) Universal Preprocessing and Feature Standardization: The sensitive nature of radiomic descriptors to scanner variation demands the prioritized development of automated, cross-vendor image harmonization software that guarantees plug-and-play feature reproducibility. (3) Multi-Omics Blueprint Expansion: Research remains siloed within pure computer vision; a significant gap exists in linking these macroscopic radiomic phenotypes to microscopic transcriptomic, proteomic, and urinary steroid metabolomic signatures. Prioritizing this multi-omics data integration will unlock the creation of truly comprehensive, high-fidelity intelligent decision support networks.

## 8 Conclusion

The integration of radiomics and DL into the evaluation of adrenal masses represents a transformative advancement in medical imaging and precision medicine. From an expert perspective, these technologies have unlocked the potential to extract intricate, high-dimensional data from routine imaging modalities—data that surpasses the perceptual limits of human observers. This capability has opened new avenues for improving differential diagnosis, genotype prediction, and prognostic assessment in adrenal pathology, thereby promising to enhance clinical decision-making and patient outcomes (19, 20).

Despite the encouraging progress demonstrated in recent studies, the field remains at a critical juncture. Current research efforts have illuminated the immense promise of radiomics and DL but have also exposed significant challenges that must be addressed to realize their full clinical utility. Among these challenges, the lack of standardized imaging protocols and data harmonization stands out as a fundamental barrier. Variability in image acquisition parameters and preprocessing techniques can lead to inconsistent feature extraction, undermining the reproducibility and reliability of predictive models (19, 41). Furthermore, the “black box” nature of many DL algorithms limits interpretability, which is a crucial factor for clinical acceptance and regulatory approval. Clinicians require transparent models that not only perform well but also provide understandable rationale for their predictions to foster trust and facilitate informed decision-making.

Another critical limitation is the generalizability of existing models. Many studies rely on retrospective, single-center datasets with limited sample sizes, which restricts the robustness of the models when applied to diverse patient populations or imaging platforms. The scarcity of large-scale, prospective, multicenter validation studies hampers the translation of these promising tools from research settings into routine clinical practice. Additionally, the integration of radiomic features with genomic data and clinical outcomes remains in its infancy. While preliminary findings suggest that combining imaging phenotypes with molecular and clinical information can enhance predictive accuracy, comprehensive multi-omics studies are needed to fully elucidate these complex relationships and to develop integrated models that reflect the multifactorial nature of adrenal diseases.

Looking forward, the future development of radiomics and DL in adrenal mass evaluation should prioritize several key areas. Enhancing the robustness and reproducibility of radiomic features through standardized imaging acquisition and preprocessing protocols is paramount. Advances in algorithmic transparency and explainability will be essential to bridge the gap between computational predictions and clinical interpretability. Emerging techniques such as attention mechanisms, saliency mapping, and model-agnostic interpretability tools hold promise in this regard. Addressing data scarcity through innovative learning paradigms—such as transfer learning, federated learning, and synthetic data augmentation—can improve model performance and generalizability without compromising patient privacy.

Moreover, deepening the integration of imaging data with genomics and clinical endpoints will be critical to developing comprehensive predictive models that can guide personalized therapeutic strategies. This necessitates collaborative efforts to build large, high-quality, annotated databases that encompass multimodal data, enabling robust training and validation of complex models. Importantly, the path to clinical translation demands a multidisciplinary approach (39, 40). Radiologists, endocrinologists, surgeons, pathologists, and bioinformaticians must work synergistically to establish standardized workflows, quality control measures, and validation frameworks. Such collaboration will facilitate the design of prospective clinical trials that rigorously assess the diagnostic and prognostic value of radiomics and DL tools in real-world settings.

In conclusion, radiomics and DL have ushered in a new era of precision assessment for adrenal masses, offering unprecedented opportunities to enhance diagnostic accuracy and prognostication. However, balancing the enthusiasm for these innovative technologies with a critical appraisal of their current limitations is essential. Through concerted multidisciplinary collaboration, methodological rigor, and comprehensive validation, the field can overcome existing challenges and translate these powerful tools into clinical practice. Ultimately, this will enable the realization of intelligent, personalized management of adrenal diseases, improving patient care and outcomes in a manner that is both scientifically sound and clinically meaningful.
